# Effects of Natural Product-Derived Compounds on Inflammatory Pain via Regulation of Microglial Activation

**DOI:** 10.3390/ph16070941

**Published:** 2023-06-29

**Authors:** Joon Park, Changho Lee, Yun Tai Kim

**Affiliations:** 1 Division of Functional Food Research, Korea Food Research Institute, Wanju 55365, Republic of Korea; biosciencepark@gmail.com (J.P.); chang@kfri.re.kr (C.L.); 2 Department of Food Biotechnology, Korea University of Science and Technology, Daejeon 34113, Republic of Korea; 3 Department of Anesthesiology, College of Medicine, The University of Arizona, Tucson, AZ 85724, USA

**Keywords:** natural products, inflammatory pain, microglial activation, microglia, neuroinflammation

## Abstract

Inflammatory pain is a type of pain caused by tissue damage associated with inflammation and is characterized by hypersensitivity to pain and neuroinflammation in the spinal cord. Neuroinflammation is significantly increased by various neurotransmitters and cytokines that are expressed in activated primary afferent neurons, and it plays a pivotal role in the development of inflammatory pain. The activation of microglia and elevated levels of pro-inflammatory cytokines are the hallmark features of neuroinflammation. During the development of neuroinflammation, various intracellular signaling pathways are activated or inhibited in microglia, leading to the regulation of inflammatory proteins and cytokines. Numerous attempts have been conducted to alleviate inflammatory pain by inhibiting microglial activation. Natural products and their compounds have gained attention as potential candidates for suppressing inflammatory pain due to verified safety through centuries of use. Many studies have also shown that natural product-derived compounds have the potential to suppress microglial activation and alleviate inflammatory pain. Herein, we review the literature on inflammatory mediators and intracellular signaling involved in microglial activation in inflammatory pain, as well as natural product-derived compounds that have been found to suppress microglial activation. This review suggests that natural product-derived compounds have the potential to alleviate inflammatory pain through the suppression of microglial activation.

## 1. Introduction

The International Association for the Study of Pain (IASP) describes pain as an unpleasant sensory and emotional experience associated with, or resembling that associated with, actual or potential tissue damage. If pain persists for more than 3 months, which is considered the tissue healing period, it is diagnosed as chronic pain. In 2019, approximately 20% of adults in the US were diagnosed with chronic pain, and the number of patients with chronic pain is increasing with the increase in the aging population [1]. According to a National Institute of Health (NIH) report, the cost of treating chronic pain exceeds that of a few major diseases related to the highest morbidity and mortality, such as cardiovascular diseases (USD 309 billion), cancers (USD 243 billion), and injuries (USD 205 billion) [2]. Patients with chronic pain have a poor quality of life for reasons such as difficulty engaging in daily activities and prolonged treatment. Patients with chronic pain also suffer from mental disorders such as depression, anxiety disorders, and sleep disturbances [3]. As the average lifespan continues to increase, the importance of pain management is also increasing.

Pain perception is a complex and highly orchestrated process involving a series of sequential events [4]. Following inflammation-related tissue damage, nociceptors, which are sensory neurons responsible for detecting harmful stimuli, initiate a response. Nociceptors convert stimuli into electrical signals, which are then transmitted to the central nervous system (CNS). Subsequently, these electrical signals are transmitted to secondary afferent neurons located in the dorsal horn of the spinal cord. With the repetitive transmission of pain signals, neuroinflammation is strongly induced within the spinal cord. Neuroinflammation profoundly influences synaptic transmission, thereby contributing to the persistence of pain.

The causes of chronic pain include nerve injury, cancer, muscle injury, and inflammation [5]. Among the various causes, pain caused by inflammation is called inflammatory pain. Currently, non-steroidal anti-inflammatory drugs (NSAIDs) are the most commonly used drugs to treat inflammatory pain [6]. The main mechanism underlying the analgesic and anti-inflammatory effects of NSAIDs is inhibition of the cyclooxygenase (COX2) enzyme, which produces prostaglandins. Prostaglandin is a representative inflammatory mediator that induces fever, inflammation, and pain. Aspirin, naproxen, and ibuprofen are some of the commonly used NSAIDs. However, since NSAIDs have adverse effects, such as indigestion, stomach ulcers, headaches, drowsiness, and dizziness, interest in natural products as agents for alleviating inflammatory pain has recently increased. Natural products have been used for centuries to treat various diseases related with inflammation, without causing side effects [7]. Therefore, natural products have the potential to be developed into new drugs against inflammatory pain. 

In this review, we aim to summarize the current understanding of inflammatory factors and intracellular signaling involved in the development of inflammatory pain as well as to highlight the potential of natural products in treating inflammatory pain.

## 2. Mechanism Underlying the Development of Inflammatory Pain

Inflammatory pain is characterized by a heightened sensitivity to pain due to tissue damage resulting from an inflammatory or immune response. The two typical symptoms of pain are hyperalgesia and allodynia. Hyperalgesia is characterized by an abnormally increased sensitivity to pain and an extreme response to pain. Allodynia is recognized as pain for common stimuli that do not normally cause pain. Chronic inflammatory diseases and infections, such as arthritis, shingles, and tissue injury are representative causes of inflammatory pain [8]. Inflammatory mediators are released locally by immune cells at the site of inflammation and can directly activate sensory neurons in peripheral tissues. Activated sensory neurons then release neuropeptides, such as substance P, calcitonin gene-related peptide, and prostanoids, into the dorsal horn of the spinal cord. Repetitive and persistent stimulation of sensory neurons can lead to the over-release of neuropeptides, resulting in neuroinflammation of the spinal cord.

**Figure 1:**
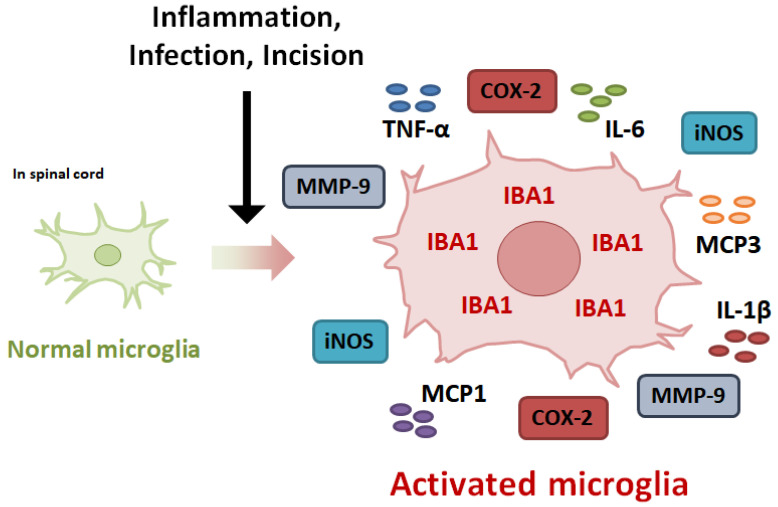
Inflammatory mediators associated with the development of microglial activation-mediated inflammatory pain. Tissue damage associated with inflammation lead to microglial activation by releasing diverse signaling molecules from sensory neurons. Activated microglia induce neuroinflammation through increasing the expression of inflammatory mediators, such as excessive NO, iNOS, COX-2, TNF-α, IL-1β, IL-6, MCP1, and MCP3. These mediators lead to central sensitization, resulting in increased sensitivity to pain. Activated microglia can trigger inflammatory pain via inflammatory mediator-related signaling.

Spinal neuroinflammation, caused by peripheral inflammation, is characterized by the activation of microglia and increased expression of inflammatory mediators in the spinal cord [9]. Activated microglia are major sources of pro-inflammatory cytokines and inflammation-related proteins that are regulated by various intracellular signaling (Figure 1). Along with spinal neuroinflammation, microglial activation is significantly induced, resulting in pain hypersensitivity through central sensitization. Central sensitization, a leading cause of chronic pain, represents the reinforcement of the function between pre- and post-synaptic neurons in the nociceptive pathway caused by increasing excitatory transmission and strengthening of synapses in response to inflammation and nerve injury. The increased levels of pro-inflammatory cytokines can enhance synaptic transmission by increasing excitatory synaptic transmission and decreasing inhibitory synaptic transmission in the dorsal horn of spinal cord [10,11]. Pro-inflammatory cytokines may induce gene expression by activating cAMP response element-binding protein (CREB) transcription factors, leading to long-term potentiation [12]. These results comprehensively contribute to the persistence and hypersensitivity of pain through central sensitization.

Microglia, the resident macrophage-like cells located in the CNS, play an important role in the development of chronic pain associated with neuroinflammation [13,14]. As the first immune cell in the CNS, microglia are essential for brain maintenance and homeostasis as they are involved in removing cell debris, infectious agents, and other unnecessary elements. Although the primary function of microglia is to protect the CNS, they can have destructive effects on neurons. Various signaling molecules are released from sensory neurons damaged by inflammation and eventually activate microglia [13]. Microglial activation is characterized by the increased production of inflammatory mediators, such as iNOS, COX-2, MMP-9, TNF-α, IL-1β, IL-6, MCP1, and MCP3. The expression of these inflammatory mediators is regulated by intracellular signaling pathways, including NF-B, MAPK, JAK2-STAT3, Nrf2, and autophagy. The expression and secretion of inflammatory mediators are induced by intracellular signaling in activated microglia, resulting in an increase in neuroinflammation [15]. Inflammatory mediators contribute to increased neuroinflammation to damage to cells. Furthermore, these mediators and cytokines are involved in the induction and maintenance of central sensitization by upregulating the NMDA receptor in excitatory synaptic neurons [16]. In agreement with these findings, pro-inflammatory cytokines, such as TNF-α, IL-1β, or IL-6, modulate the function of receptors associated with central sensitization in the spinal cord [12]. Experimental studies using rodent models have shown that intrathecal injection of pro-inflammatory cytokines induces pain hypersensitivity [10,17,18]. A previous study showed that the specific deletion of microglia in the spinal cord had an inhibitory effect on formalin-induced inflammatory pain via the modulation of central sensitization [19]. Inhibition of microglial activation using chemogenetic approaches, specifically DREADD, alleviate neuroinflammation and chronic pain following nerve injury [20]. Inhibition of microglial activation attenuated nerve injury-induced pain hypersensitivity in the early phase but not the late phase. Therefore, understanding and targeting the processes and factors involved in microglial activation-induced neuroinflammation may offer an effective approach to prevent the early phase of inflammatory pain, which has the potential to become chronic pain.

## 3. Expression of Inflammatory Mediators in Activated Microglia

### 3.1. Inducible Nitric Oxide Synthase

Nitric oxide synthase (NOS) is an enzyme that catalyzes the production of nitric oxide (NO) from L-arginine. Among the different isoforms of NOS, inducible nitric oxide synthase (iNOS) plays a significant role in the development of inflammatory pain. iNOS is induced in various cells and tissues by cytokines and other molecules. Although previous studies have reported the role of iNOS in various inflammatory diseases except for the CNS, it has been recently confirmed that iNOS contributes to the development of chronic pain [21,22]. iNOS continuously produces large amounts of NO until it is degraded. High amounts of NO result in the production of high levels of reactive nitrogen oxide species (RNOS), causing damage to the surrounding tissue and cells. Among various cells in the CNS, microglia are the major cellular sources of iNOS [23]. Many previous studies have shown that activated microglia remarkably increase the expression of iNOS, leading to the excessive production of NO [24,25,26]. Additionally, iNOS expression and neuroinflammation in the dorsal horn of the spinal cord were found to have significantly increased in CFA-injected mice [27]. Osborne et al. reported that carrageenan-induced thermal hyperalgesia was significantly alleviated by the intrathecal injection of the nonselective NOS inhibitor L-NAME in rats. Furthermore, a selective iNOS inhibitor suppressed thermal hypersensitivity during carrageenan-induced inflammatory pain [28]. Formalin-induced pain behavior was attenuated in iNOS knockout mice. Moreover, nerve injury-induced pain hypersensitivity and microglial activation in the spinal cord were suppressed in the iNOS knockout mice compared with in wild-type mice [26]. These results demonstrated that iNOS expression in activated microglia can exacerbate neuroinflammation, resulting in increased pain sensitivity.

### 3.2. Cyclooxygenase-2

Cyclooxygenase-2 (COX-2) is a primary target for reducing inflammation and pain. COX-2 converts arachidonic acid to prostaglandin E2 (PGE2), which is associated with inflammation and pain. COX-2 is induced in response to inflammatory stimulation and is primarily expressed by monocytes, macrophages, fibroblasts, neurons, and microglia [29,30]. COX-2 inhibition is expected to reduce inflammation and pain without causing side effects [31]. Many studies have shown that lipopolysaccharide (LPS) treatment increased COX-2 expression in microglia [32,33,34,35]. These studies suggest that neuroinflammation can be suppressed by inhibition of COX-2 expression in microglia. In addition, various COX-2 inhibitors suppressed neuroinflammation by inhibiting the release of inflammatory mediators by microglia [36]. Naproxen is a representative oral NSAID; it attenuated CFA-induced pain hypersensitivity by inhibiting COX-2 expression in the spinal cord [37]. Therefore, microglia-specific inhibition of COX-2 expression has the potential to alleviate inflammatory pain.

### 3.3. Matrix Metalloproteinases-9

Matrix metalloproteinases-9 (MMP-9) is a member of the zinc metalloproteinase family involved in the degradation of the extracellular matrix and is strongly implicated in the development of various neuroinflammation-related diseases [38]. Microglia are a major source of MMP-9. Nerve injury has been found to increase the expression of MMP-9 rapidly and temporarily in the dorsal root ganglion (DRG), leading to the induction of neuropathic pain [39,40]. Microglial activation is also increased by nerve injury-induced MMP-9 expression in the spinal cord. Intrathecal injection of siMMP-9 significantly attenuated nerve injury-induced pain hypersensitivity and inhibited microglial activation in the spinal cord. A previous study found that activated microglia significantly increased the expression of MMP-9 in LPS-treated microglial cells [41]. Additionally, in one study, MMP-9 expression was notably upregulated in the DRG and spinal cord in a CFA-induced inflammatory pain model [42]. This study showed that inhibition of MMP-9 had inhibitory effects on CFA-induced pain hypersensitivity in rats. These studies indicated that MMP-9 is involved in the development of microglial activation-mediated inflammatory pain.

### 3.4. Pro-Inflammatory Cytokines

Cytokines are secreted mainly by the immune and glial cells of the CNS. These cytokines act as intercellular mediators that control the function and differentiation of other cells [43]. In response to peripheral inflammation and tissue injury, microglia can be activated to secrete pro-inflammatory cytokines, such as TNF-α, interleukin-1β (IL-1β), IL-6, monocyte chemotactic protein-1 (MCP1), and MCP3 [24,33,34,37]. These pro-inflammatory cytokines are strongly involved in neuroinflammation and mainly contribute to the exacerbation of chronic pain.

#### 3.4.1. TNF-α

Tumor necrosis factor-α (TNF-α) is a cytokine that causes inflammation-related diseases and might be a potential therapeutic target. Moreover, TNF-α is proposed to be a pro-inflammatory cytokine that plays a critical role in the development of chronic pain [43]. Microglia express and secrete TNF-α in response to stimuli and can also be activated by TNF-α via TNF receptors (TNFRs) [44]. In one study, TNF-α induced microglial activation, as evidenced by the increased expression of iNOS, IL-1β, and IL-6 in primary cells [45]. This study showed that LPS-induced microglial activation was partially blocked by treatment with TNFR type 1 (TNFR1) antibody. Moreover, intrathecal injection of TNF-α significantly induced pain hypersensitivity in mice. In addition, TNFR1 knockout mice exhibited better inhibition of pain hypersensitivity than TNFR2 knockout mice in CFA and formalin-induced inflammatory pain models [10]. Thus, TNF-α can activate microglia via binding to TNFR1 to increase pain sensitivity.

#### 3.4.2. Interleukin-1β

Interleukin-1β (IL-1β) is one of the important mediators of the inflammatory response and is implicated in microglial activation-mediated inflammatory pain. The IL-1β precursor is cleaved by cytosolic caspase 1 and activated to mediate the inflammatory response. Previous studies showed that IL-1β could activate microglia, as revealed by the increased expression of IL-6, MCP1, and CXCL10 in human microglia cells [46,47]. IL-1β is a major mediator that increases the expression of COX-2 in the spinal cord, resulting in the development of CFA-induced inflammatory pain [48]. In one study of a mouse model, inhibition of IL-1β in the spinal cord decreased sensitivity to pain by decreasing the expression of COX2. Another study reported that intravenous injection of IL-1β significantly increased pain hypersensitivity and microglial activation in dorsal horn of the spinal cord [49].

#### 3.4.3. Interelukin-6

Interelukin-6 (IL-6) is also a well-known inflammatory cytokine along with TNF-α and IL-1β. IL-6 is an important mediator of fever and pathogenesis of chronic pain. In one study, exposure to LPS remarkably induced the expression of IL-6 in microglia [50]. Furthermore, formalin- and CFA-injected mice showed notably increased microglial activation and IL-6 expression in the spinal cord [51,52]. In addition, nerve injury-induced IL-6 expression was decreased by microglia inhibitor in serum and spinal cord [17,53]. This study showed that intrathecal injection of IL-6 significantly increased microglial activation in the spinal cord. These data indicate the presence of a positive feedback loop between IL-6 and microglial activation, resulting in the pathogenesis of microglial activation-mediated inflammatory pain.

#### 3.4.4. Monocyte Chemoattractant Protein-1

MCP1 is one of the key chemokines that control the migration and infiltration of monocyte. MCP1 plays an important role in the development of chronic pain. Although MCP1 is known to interact with several receptors, C-C chemokine receptor type 2 (CCR2) is its preferred receptor [54]. Direct injection of MCP1 into the spinal cord induced pain hypersensitivity, whereas co-treatment with an MCP1 inhibitor reduces sensitivity to pain by blocking central sensitization [11]. This study showed that CFA-induced pain hypersensitivity in mice was significantly attenuated by intrathecal injection of a CCR2 inhibitor. Additionally, the intrathecal injection of MCP1 induced microglial activation in the spinal cord [55]; spinal microglial activation was also markedly decreased by an antibody against MCP1. A previous study reported that MCP1 was strongly increased in DRG neurons due to peripheral inflammation and was transported into the spinal cord [56]. In addition, neuron-derived MCP1 notably induced microglial activation, as evidenced by the upregulated expression of iNOS, COX2, IL-1β, and IL-6 [57]. Several in vitro experiments showed that activated microglia could secrete MCP1 [24,58]. These results suggest that peripheral inflammation induces MCP1 expression in the neurons. MCP1 is transported into the spinal cord, followed by autocrine activation of microglia by MCP1. Therefore, peripheral inflammation-induced MCP1 expression may increase pain sensitivity by activation of microglia through a positive feedback loop.

#### 3.4.5. Monocyte Chemoattractant Protein-3

MCP3 is a small cytokine that is closely related to MCP1. MCP3 has been found to play a role in the development of chronic pain. In one study, nerve injury remarkably induced MCP3 expression in the spinal cords of mice [18]. Moreover, nerve injury-induced microglial activation was decreased in the spinal cord of CCR2 knockout mice compared to in normal mice. Moreover, intrathecal injection of MCP3 induced pain hypersensitivity in a dose-dependent manner, while intrathecal injection of a CCR2 inhibitor or antibody against MCP3 reduced sensitivity to pain in mice. This study suggests that MCP3 is primarily expressed in astrocytes. Astrocyte-derived MCP3 plays a key role in the development of neuropathic pain. However, many studies have reported that MCP3 is also expressed in activated microglia [24,59]. Furthermore, CFA-induced pain hypersensitivity was attenuated by inhibition of microglial activation and MCP3 expression in the spinal cord. Microglial activation was inhibited by MCP3 knockdown. Therefore, inhibition of MCP3 expression may alleviate inflammation-induced pain hypersensitivity by regulating microglial activation in the spinal cord.

## 4. Intracellular Signaling in Activated Microglia

**Figure 2:**
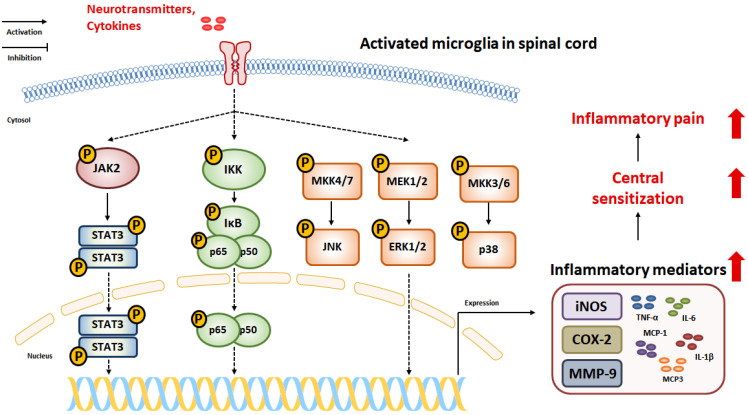
Intracellular signaling that can induce inflammatory mediators in activated microglia. NF-κB, MAPK, and JAK2/STAT3 signaling can be activated in microglia. And this signaling can induce expression of inflammatory mediators in activated microglia, leading to exacerbation of inflammatory pain.

Activated microglia release inflammatory mediators that may contribute to hypersensitivity to inflammatory pain. Many studies have demonstrated the importance of intracellular signaling pathways that are strongly involved in microglial activation-mediated inflammatory pain. Activation of microglia leads to the induction of cascades of numerous intracellular signaling pathways. These signaling pathways may contribute to changes in the function of microglia, and gene expression resulting from these signaling pathways may influence the functions and structures of nearby cells and tissues, resulting in exacerbated inflammatory pain. Understanding how intracellular signaling pathways work in activated microglia may help identify new therapeutic targets for inflammatory pain (Figure 2).

### 4.1. Nuclear Factor-κB

Nuclear factor-κB (NF-κB) is a representative of the family of transcript factors associated with the inflammatory response. The activation of NF-κB signaling involves two major pathways: canonical and noncanonical. Although both pathways are important for regulating the inflammatory response, the noncanonical pathway is particularly involved in regulating specific functions of the adaptive immune system [60]. This chapter focuses on canonical NF-κB signaling, which is associated with microglial activation. Canonical NF-κB signaling is triggered by various stimuli [61]. The first step in the canonical NF-κB signaling pathway is the activation of the IκB kinase (IKK) complex, which comprises IKKα, IKKβ, and IKKγ subunits [62]. The IKK complex phosphorylates IκBα, leading to ubiquitylation and proteasomal degradation. This results in the phosphorylation and nuclear translocation of the NF-κB dimer (p65 and p50). The translocated NF-κB dimer binds to a specific DNA sequence and promotes the transcription of target genes. NF-κB signaling is strongly associated with the development of microglial activation, as evidenced by increased expression of inflammatory mediators and cytokines [63]. Previous studies have shown that microglial activation was significantly reduced by treatment with NF-κB inhibitors [64,65]. Moreover, LPS-induced expression of inflammatory cytokines and mediators was reduced by suppression of NF-κB activity through IKK-specific deletion in microglia [66]. Inhibition of NF-κB in the spinal cord showed an alleviative effect on CFA-induced pain hypersensitivity and microglial activation [67]. Furthermore, nerve injury-induced upregulation of pain sensitivity was alleviated by the suppression of NF-κB signaling in microglial activation [68]. These data demonstrate that activation of NF-κB signaling in microglia can lead to increased pain sensitivity in inflammatory pain.

### 4.2. Mitogen-Activated Protein Kinase

The mitogen-activated protein kinase (MAPK) signaling pathway, which comprises the c-Jun N-terminal kinase (JNK), extracellular signal-regulated kinase (ERK), and p38 mitogen-activated protein (p38) kinase, plays a crucial role in regulating various cellular functions such as proliferation, differentiation, development, and migration [69]. MAPK is involved in protein kinase cascades, in which they are activated in a sequential manner by the upstream signals such as MAPKK and MAPKKK. 

In the nervous system, JNK is implicated in the pathogenesis of various neuroinflammation-related diseases [70,71]. JNK is activated by its upstream signals, MKK4 and MKK7, leading to the phosphorylation of the downstream signal, c-Jun. The activated JNK could induce the production of inflammatory mediators and cytokines in the CNS. Thus, the inhibition of JNK has been considered a therapeutic target for the treatment of neurodegenerative diseases. In addition, the inhibition of JNK in microglia has been suggested to attenuate inflammatory pain. Previously, LPS-induced microglial activation was remarkably suppressed by treatment with a JNK inhibitor, as revealed by the reduced expression of inflammatory mediators and cytokines [72]. Additionally, CFA-induced pain hypersensitivity was attenuated by intrathecal injection of a JNK inhibitor in rats [73].

Among the MAPK family members, ERK1/2 activation by MEK1/2 is generally considered to regulate cell survival, proliferation, and differentiation. The activation of ERK in microglia leads to neuroinflammation by increasing the expression of pro-inflammatory cytokines and inflammatory mediators [74]. These effects have become the cornerstone in the development of neurodegenerative diseases. A previous study identified that ERK activation in microglia was significantly increased in the early stages of nerve injury-induced chronic pain [75]. In in vitro experiments, LPS-induced expression of iNOS, COX-2, and pro-inflammatory cytokines was notably reduced by treatment with ERK inhibitor in microglia [76,77]. In addition, direct injection of ERK inhibitor into the spinal cord showed inhibitory effects on CFA-induced pain hypersensitivity in mice [78,79]; these effects were accompanied by a reduction in COX-2 expression in the spinal cord. 

p38 is more strongly involved in the development of chronic pain related to microglial activation than other MAPK family members. p38 is activated by the upstream kinases MKK3 and MKK6 and plays an important role in the inflammatory response. p38 inhibitors have been found to alleviate inflammatory diseases [80]. During the development of chronic pain, p38 activation is notably increased in microglia compared to that in other cells [81]. In addition, the activation of p38 in microglia was upregulated in CFA-induced inflammatory pain. Moreover, intrathecal injection of p38 inhibitor attenuated CFA- or formalin-induced pain-like behaviors and significantly suppressed expression of IL-1β and IL-6 in the spinal cord [82,83,84]. In in vitro studies, LPS-induced NO overproduction and expression of iNOS and COX2 were reduced by treatment with a p38 inhibitor in a dose-dependent manner [37]. Taken together, these results demonstrate that MAPK signaling is critical for microglial activation and leads to the pathogenesis of inflammatory pain.

### 4.3. Janus Kinase 2 (JAK2)/Signal Transducer and Activator of Transcription 3

The Janus kinase 2 (JAK2)/signal transducer and activator of transcription 3 (STAT3) pathway is an intracellular signaling pathway activated by cytokines. The JAK2/STAT3 pathway is involved in immune cell division, development, recruitment, and activation. Many studies have indicated that microglial activation is dependent on the phosphorylation of the JAK2/STA3 signaling pathway caused by various stimuli [85,86,87]. These data revealed that inhibition of JAK2/STAT3 signaling suppressed microglial activation, as shown by the decreased expression of inflammatory cytokines and mediators. Additionally, CFA-induced pain hypersensitivity and spinal microglial activation were significantly reduced by inhibition of the JAK2/STAT3 signaling pathway in rodents [24,88,89]. These results indicate that JAK2/STAT3 signaling plays an important role in microglial activation and development of inflammatory pain.

### 4.4. Nuclear Factor-Erythroid 2-Related Factor 2

**Figure 3:**
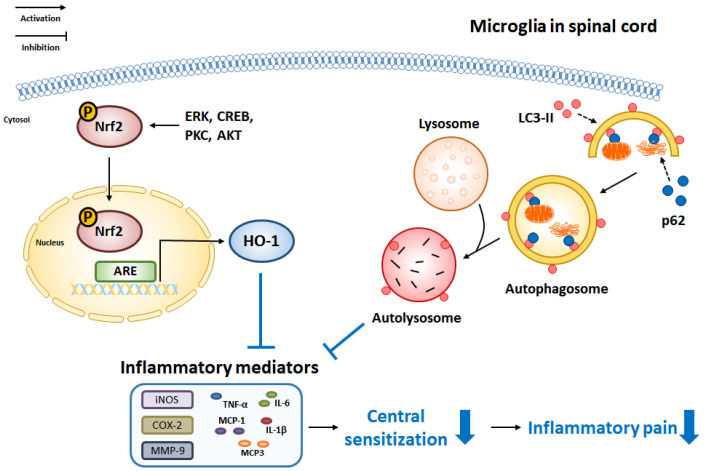
Intracellular signaling that can regulate inflammatory mediators in microglia. Nrf2 signaling and autophagy have a protective effect on microglial activation. Inflammatory mediators expressed in activated microglia are suppressed by activation of Nrf2 signaling and autophagy.

Nuclear factor-erythroid 2-related factor 2 (Nrf2) is a transcription factor that regulates antioxidant enzymes to protect against damage caused by oxidative stress. Oxidative stress is a hallmark of neuroinflammation and neurodegeneration that leads to disease progression [90]. Accumulating evidence has shown that microglial activation was significantly increased in the CNS of Nrf2-deficient mice, contributing to the exacerbation of neurodegenerative diseases (Figure 3) [91,92]. Previous studies have shown that microglial activation was increased in Nrf2 knockout mice, as revealed by the upregulated expression of pro-inflammatory cytokines and inflammatory mediators [93,94]. Nrf2 induces gene expression via interaction with an antioxidant response element (ARE) that is known to encode antioxidant enzymes. Nrf2-dependent gene expression exerts a protective effect against oxidative stress in microglia, resulting in the suppression of neuroinflammation. Moreover, neuroinflammation-mediated chronic pain is regulated by the activation of Nrf2 in microglia. Among Nrf2-dependent genes, the heme oxygenase-1 (*HO-1*) gene is a representative gene with strong antioxidant effects. Previously, Nrf2-dependent HO-1 expression in microglia showed inhibitory effects against CFA-induced pain hypersensitivity and microglial activation in mice [75]. Additionally, the administration of an HO-1 inducer significantly attenuated formalin-induced pain-like behavior in mice. However, the attenuative effect of the HO-1 inducer was reversed in Nrf2-knockout mice [95]. These results indicated that Nrf2 inhibits inflammation-induced microglial activation and pain hypersensitivity via HO-1 induction.

### 4.5. Autophagy

Autophagy is a lysosomal degradation pathway responsible for the removal and recycling of unnecessary or dysfunctional molecules maintain cellular homeostasis [96]. The process begins by the marking of unwanted or damaged molecules for removal, followed by the formation of an autophagosome that envelopes the unwanted molecules. This autophagosome then combines with a lysosome to degrade the cargo, after which unwanted molecules are removed and recycled. Autophagy has a protective effect, as demonstrated by its ability to remove amyloid-β, a hallmark of Alzheimer’s disease, and prevent neurodegeneration in mice [97]. Additionally, microglial autophagy has been found to play a role in regulating neuroinflammation. LPS-treated microglia showed inhibited autophagic activity, leading to increased neuroinflammation. However, treatment with the autophagy inducer, rapamycin, significantly reduced LPS-induced neuroinflammation in microglia [98]. CFA-induced pain hypersensitivity was attenuated by the induction of autophagy in the spinal cord [24,99,100]. In addition, CFA-induced expression of pro-inflammatory cytokines and microglial activation were decreased by enhanced autophagy in the spinal cord. These results suggested that autophagy activation may have a protective effect against microglial activation and inflammatory pain.

## 5. Natural Product-Derived Compounds against Microglial Activation-Mediated Inflammatory Pain

**Figure 4:**
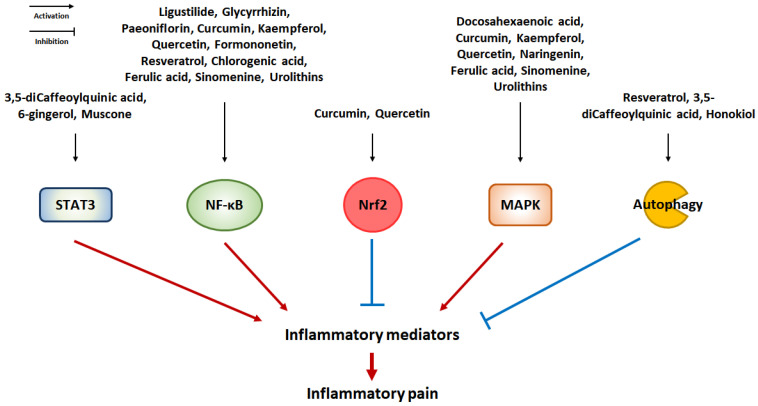
Effect of natural product-derived compounds on inflammatory pain via the suppression of microglial activation. Natural products and phytochemicals may have suppressive effects on microglial activation. Each compound may inhibit microglial activation by regulating the intracellular signaling pathways. The suppression of microglial activation by the modulation of intracellular signaling via natural product-derived compounds shows potential for attenuating inflammatory pain.

Multiple studies have demonstrated that microglial activation contributes significantly to the development of inflammatory pain. Therefore, targeting microglial activation through the regulation of inflammatory mediators has been proposed as a therapeutic strategy for the treatment of inflammatory pain. Many natural products and their compounds have been found to exert protective effects against inflammation [101]. These studies suggest that natural product-derived compounds with anti-inflammatory effects inhibit inflammatory pain by suppressing microglial activation. Table 1 and Figure 4 present a summary of natural product-derived compounds that have been found to have the potential to alleviate microglial activation-mediated inflammatory pain.

### 5.1. 3,5-Dicaffeoylquinic Acid

3,5-Dicaffeoylquinic acid (3,5-DCQA) is a phenolic nutraceutical present in *Arctium lappa* and *Aster yomena*; it has shown inhibitory effects on the LPS-induced expression of iNOS and COX-2 and secretion of TNF-α, IL-1β, IL-6, MCP1, and MCP3 in BV2 microglial cells [24]. Suppression of MCP3 expression by 3,5-DCQA enhanced autophagy by suppressing LPS-induced activation of JAK2-STAT3, resulting in the reduction of microglial activation. Furthermore, CFA-induced pain hypersensitivity was attenuated by the administration of 3,5-DCQA. Additionally, the administration of 3,5-DCQA suppressed microglial activation in the spinal cord of CFA-injected mice.

### 5.2. Chlorogenic Acid

Chlorogenic acid, an ester of caffeic and quinic acids, is a natural phenolic compound found in plants. One study showed that chlorogenic acid significantly inhibited LPS-induced NO production and expression of iNOS and TNF-α in primary microglia [102]. In addition, LPS-induced phosphorylation of NF-κB signaling was suppressed by treatment with chlorogenic acid. Carrageenan-induced foot swelling and formalin-induced pain-like behavior were significantly reduced by oral administration of chlorogenic acid in mice [103]. In a clinical study, plasma antioxidant capacity was significantly increased in the group that consumed chlorogenic acid-rich coffee/day. Participants had consumed a maximum of 480 mg/day chlorogenic acid for 8 weeks and did not experience any adverse effects [104]. Furthermore, a clinical study identified that the consumption of chlorogenic acid resulted in improved neuronal function [105]. 

### 5.3. Ferulic Acid

Ferulic acid, a well-known phenolic compound, is a bioactive compound found in medicinal herbs, including *Ferula asafoetida*. One study showed that LPS-induced expression of iNOS and TNF-α was reduced after treatment with ferulic acid in BV2 microglial cells in a dose-dependent manner [106]. Additionally, LPS-induced phosphorylation of JNK and NF-κB was reduced by ferulic acid. Moreover, formalin-induced pain-like behavior in mice was alleviated by intraperitoneal injection of ferulic acid [107]. In a clinical study, participants were administered 1000 mg/day ferulic acid for 6 weeks. No toxicity associated with this dose of ferulic acid was observed. The oxidative stress marker was significantly reduced in the group supplemented with ferulic acid. Moreover, TNF-α was remarkably reduced in blood samples [108]. Another clinical study also showed clinical positive effective of ferulic acid on neuronal functioning [109].

### 5.4. 6-Gingerol

6-gingerol, present in *Zingiber officinale*, is a bioactive phenolic compound, which is known to have a neuroprotective effect. Previously, LPS-induced NO production and expression of iNOS, IL-1β, and IL-6 were dose-dependently suppressed by treatment with 6-gingerol, leading to inhibition of microglial activation [110]. LPS-induced phosphorylation of STAT3 in microglia was significantly reduced by 6-gingerol treatment. Furthermore, intraperitoneal injection of 6-gingerol was found to attenuate acetic acid- and formalin-induced pain-like behaviors, such as writhing and licking, in mice [111]. Carrageenan-induced paw swelling was also suppressed by the administration of 6-gingerol. No clinical trials have specifically investigated analgesic effects of 6-gingerol alone. However, numerous studies have reported alleviative effects of ginger on inflammation-related pain in humans [112]. Given that 6-gingerol had no adverse effects at a concentration of 10 mg twice a day for 12 weeks, it is necessary to evaluate the analgesic effects of 6-gingerol using this dosage [113]. 

### 5.5. Curcumin

Curcumin is a bright yellow bioactive component found in *Curcuma longa*. LPS-induced expression of iNOS, TNF-α, and IL-1β was decreased after treating BV2 microglial cells with curcumin [114]. Additionally, lipoteichoic acid (LTA) treatment increased the production of NO and PGE3, and expression of iNOS, COX-2, and TNF-α, reversed by treatment with curcumin in BV2 microglial cells. In one study, curcumin showed a suppressive effect on microglial activation via inhibition of NF-κB and MAPK signaling and induction of Nrf2 in BV2 microglial cells [115]. Moreover, in another study CFA-induced hyperalgesia was attenuated via suppression of TNF-α, IL-1β, and IL-6 in the spinal cord by administration of curcumin [116]. In a clinical study identifying anti-inflammatory effects of curcumin, the reduction in pain sensitivity and inflammation at the surgical site was evaluated in a group of patients who receive 400 mg of curcumin three times a day for 6 days [117]. 

### 5.6. Kaempferol

Kaempferol, one of the most common flavonoids found in numerous medicinal herbs, is known to have antioxidant and anti-inflammatory effects. In one study, LPS-induced microglial activation was suppressed by treatment with kaempferol, as revealed by decreased production of NO and PGE2 and decreased expression of iNOS, COX-2, MMP9, TNF-α, and IL-1β in microglia [118]. The underlying inhibitory mechanism of kaempferol is the inhibition of NF-κB and MAPK signaling pathways in microglia. Moreover, formalin-induced pain hypersensitivity was alleviated by intrathecal injection of kaempferol in mice [119]. Administration of kaempferol showed an inhibitory effect on the formalin-induced expression of TNF-α, IL-1β, and IL-6 in the spinal cord. A variety of clinical studies had provided evidence for the preventive effects of kaempferol on diseases associated with inflammation [120,121]. The consumption of 50 mg a day of kaempferol for 4 weeks is safe in adults [122]. 

### 5.7. Quercetin

Quercetin is considered an antioxidant, anti-inflammatory, and anti-nociceptive compound. Studies have shown that quercetin inhibits LPS-induced NO production and iNOS expression in BV2 microglial cells. Furthermore, LPS-induced NF-κB activation was reduced by quercetin treatment [123]. Additionally, in one study, quercetin led to the activation of Nrf/HO-1 signaling, resulting in the inhibition of NO production in microglia. Moreover, CFA-induced chronic inflammatory hyperalgesia was attenuated by the inhibition of ERK1/2 and NF-κB in the spinal cord after the administration of quercetin [124]. CFA-induced TNF-α expression was also decreased in the spinal cord by quercetin administration. A previous study investigated the effect of quercetin supplementation on inflammation and pain in women diagnosed with rheumatoid arthritis [125]. The patients were given 500 mg of quercetin a day for 8 weeks. The results revealed significant reductions in plasma levels of TNF-α and improvements in symptoms related to swelling and pain in patients following quercetin supplementation. Notably, no side effects were observed in the patients.

### 5.8. Formononetin

Formononetin is a bioactive isoflavone found in various plants including *Trifolium pratense* L. In one study, LPS-induced microglial activation was reduced by treatment of formononetin, as revealed by a decrease in the expression of TNF-α, IL-1β, and IL-6 [126]. Additionally, LPS-induced expression of iNOS and COX-2 was suppressed in BV2 microglial cells. Moreover, formononetin showed an inhibitory effect on LPS-induced activation of NF-κB signaling. In the CFA-induced inflammatory pain model, the administration of formononetin alleviated mechanical allodynia and thermal hyperalgesia in mice [127]. Formononetin has been studied in preclinical tests for other diseases, but clinical studies for the use of formononetin alone have yet to be performed. A previous study showed that extracts containing rich-formononetin (50 mg/day for at least 1 year) exhibited beneficial effects on the bone, with no significantly adverse effects [128]. However, further clinical research is required to determine the safety and effects of formononetin specifically on inflammatory pain. 

### 5.9. Naringenin

Naringenin is a flavonoid with antioxidant, anti-inflammatory, and anti-cancer properties. One study revealed that naringenin blocked transformation into LPS-induced activation, as evidenced by expression of iNOS, TNF-α, and IL-1β in BV2 microglia cells [129]. In addition, the LPS-induced phosphorylation of MAPK members, including JNK, ERK, and p38, was notably inhibited by naringenin treatment. In mice with inflammatory pain, carrageenan-, capsaicin-, CFA-, and PGE2-induced mechanical hyperalgesia was significantly alleviated by the oral administration of naringenin without gastric or hepatic toxicity [130]. A study conducted on healthy adults to evaluate the safety and pharmacokinetics of naringenin reported that the half-life was 3 h and almost disappeared from the serum after 24 h of ingestion [131]. No adverse events were reported up to 900 mg of naringenin. Clinical trials using orange juice, which is known to contain naringenin, showed anti-inflammatory effects as evidenced by increasing pro-inflammatory cytokines [132]. These findings suggest that naringenin has the potential to attenuate pain through the regulation of inflammation.

### 5.10. Resveratrol

Resveratrol is a bioactive component produced in grapes and is a representative inducer of autophagy. One study reported that LPS/interferon γ (IFNγ)-induced expression of iNOS, TNF-α, and IL-1β was suppressed by resveratrol treatment in N9 microglial cells [133]. In the case of LPS/IFNγ-induced microglial activation, activation of NF-κB was inhibited by resveratrol treatment. Moreover, in one study, CFA-induced temporomandibular disorders, resveratrol dose-dependently attenuated pain-like behavior in mice [134]. TNF-α in activated microglia of spinal trigeminal nucleus caudalis is also inhibited by resveratrol treatment. In a clinical study, resveratrol was evaluated for its effects on inflammation and pain in patients with knee osteoarthritis [135]. A total of 110 patients were treated with 500 mg/day resveratrol for 90 days. In the group that received oral administration of resveratrol, pain sensitivity and pro-inflammatory cytokines in serum were significantly decreased compared to the control group. 

### 5.11. Honokiol

Honokiol, a natural polyphenolic compound, is extracted from the bark and seeds of *Magnolia officinalis*. Honokiol is an autophagy inducer that suppresses skin cancer [136]. LPS-induced NO production and expression of iNOS, IL-1β, and IL-6 in primary microglia were suppressed after treatment with honokiol [137]. In addition, in one study, carrageenan- and CFA-induced mechanical hyperalgesia, allodynia, and thermal hyperalgesia were alleviated by intraperitoneal injection of honokiol in mice [138]. In a clinical study aimed at evaluating safety, 50 mg per kg of honokiol was intravenously injected into cancer patients [139]. There were no serious adverse effects and a positive clinical response was achieved in patients. Therefore, an evaluation is needed of the anti-inflammatory and analgesic effects of honokiol at the same concentration in humans. 

### 5.12. Ligustilide

Ligustilide, a major compound found in the roots of *Angelica sinensis*, has a protective effect against inflammation in microglia. Studies have shown that LPS-induced NO production and expression of iNOS and COX2 were remarkably reduced in microglia after ligustilide treatment. LPS-induced production of TNF-α, IL-1β, IL-6, and MCP1 was also suppressed by ligustilide treatment in previous studies [140,141]. Furthermore, in one study CFA-induced pain hypersensitivity and microglial activation in the spinal cord were significantly reduced after ligustilide treatment; ligustilide treatment alleviated acetic acid- and formalin-induced pain in mice [142]. Following a safety evaluation of ligustilide in rats, oral administration of 90 mg/kg ligustilide had good health status, without any histopathological change [143]. Moreover, tissue analysis indicated that ligustilide could penetrate the blood–brain barrier. Based on these results, it is suggested that ligustilide needs to be evaluated for safety and effects on inflammatory pain in clinical settings.

### 5.13. Glycyrrhizin

Glycyrrhizin, a triterpene saponin present in *Glycyrrhiza glabra*, has shown inhibitory effects on inflammatory pain by suppressing microglial activation [144]. In one study, LPS-induced microglial activation was significantly reduced by treatment with glycyrrhizin, as evidenced by decreased NO production and expression of pro-inflammatory cytokines. Glycyrrhizin inhibited LPS-induced HMGB1/TLR4/NF-κB signaling in microglia, leading to reducing microglial activation. Additionally, CFA-induced pain hypersensitivity was attenuated by the administration of glycyrrhizin in mice. Glycyrrhizin suppresses CFA-induced expression of pro-inflammatory cytokines and activation of NF-κB in the spinal cord. In a clinical study, glycyrrhizin was administered to patients with the selective serotonin reuptake inhibitor (SSRI) in order to evaluate the effects on depression and inflammation [145]. The results showed serum levels of TNF-α, and IL-1β were significantly reduced and depressive symptoms were improved. No patients experienced severe adverse events with 150 mg/3 times a day glycyrrhizin in combination with a 10 mg/day SSRI for 4 weeks.

### 5.14. Docosahexaenoic Acid

Docosahexaenoic acid (DHA) is the major bioactive omega-3 polyunsaturated fatty acid. DHA is known to regulate the inflammatory responses in neurodegenerative diseases. In one study, carrageenan-induced inflammatory pain and microglial activation were inhibited by the administration of DHA [146]. Carrageenan-induced mechanical allodynia was inhibited by intrathecal injection of DHA in mice. After DHA treatment, carrageenan-induced microglial activation was suppressed by p38 inhibition in spinal microglia. Further, LPS-induced expression of TNF-α, IL-1β, IL-6, MCP1, CCL3, and CXCL10 was significantly suppressed by DHA treatment of BV2 microglial cells. The consumption of omega-3 fatty acid showed improvements in pain and function in patients with osteoarthritis [147]. The patients who consumed 0.45 omega-3 fatty acids/day for 24 months experienced beneficial effects on osteoarthritis without any adverse events. Thus, it is necessary to evaluate the analgesic effects and safety specifically using DHA alone in clinical settings.

### 5.15. Paeoniflorin

Paeoniflorin, the main active ingredient of *Paeonia lactiflora*, is known to reduce CFA-induced pain hypersensitivity and mRNA expression of TNF-α, IL-1β, and IL-6 in the spinal cord [148]. Furthermore, in one study, CFA-induced microglial activation in the dorsal horn was inhibited by paeoniflorin. In in vitro experiments, LPS-induced pro-inflammatory cytokines were reduced by treatment with paeoniflorin via inhibiting AKT- NF-κB in microglia. In a clinical study, intravenous injection of powders containing 35.8 mg/day paeoniflorin for 7 days showed no adverse events in healthy adults [149]. 

### 5.16. Sinomenine

Sinomenine is found in *Sinomenium acutum* and is known to have various pharmacological effects such as anti-cancer, anti-inflammation, and antioxidant effects. One study found that treatment with sinomenine suppressed amyloid-β-induced microglial activation, as evidenced by the reduction in NO production and expression of TNF-α, IL-1β, and MCP1 in BV2 cells [150]. Moreover, CFA-induced pain hypersensitivity was inhibited by intraperitoneal injection of sinomenine in mice [151]. In addition, CFA-induced expression of TNF-α, IL-1β, IL-6, and COX-2 and PGE2 production was inhibited by the activation of p38 and NF-κB in the spinal cord. In a clinical study, patients with osteoarthritis were orally administered 20 mg/2 times a day sinomenine for 3 months [152]. The results indicated that disease symptoms were attenuated and plasma levels of pro-inflammatory cytokines were significantly reduced by sinomenine in patients. No adverse events were observed during the study. Based on these findings, further evaluation is necessary to assess the attenuative effects of sinomenine on inflammatory pain. 

### 5.17. Muscone

Muscone is found in musk, which is a glandular secretion of musk deer; it is a pharmacologically bioactive compound that has been used in medicine for centuries. One study revealed that muscone had an inhibitory effect on LPS-induced NO production and expression of iNOS, IL-1β, and IL-6 in BV2 microglial cells [89]. In addition, LPS-induced activation of JAK2/STAT3 signaling was significantly suppressed by muscone treatment of BV2 cells. In an inflammatory pain model, CFA-induced pain hypersensitivity was attenuated by the intraperitoneal injection of muscone in mice. Moreover, muscone administration suppressed CFA-induced expression of pro-inflammatory cytokines and phosphorylation of JAK2/STAT3 signaling in the spinal cord of mice. Muscone exhibited liver toxicity in Kunming mice at doses exceeding 50 mg/kg [153]. As a result, additional studies in pro-clinical and clinical trials are required to further investigate the safety and effects of muscone on inflammatory pain. 

### 5.18. Urolithins

Urolithins are secondary metabolites formed by gut microbiome from ellagic acid and ellagitannins found in foods like pomegranate. Urolithins suppressed LPS-induced production of NO and mRNA expression of TNF-α, IL-1β, IL-6, iNOS, and COX-2 in BV2 microglial cells. Additionally, LPS-induced activation of ERK, p38, and NF-κB signaling were significantly reduced by urolithins in BV2 cells [154]. Another group induced experimental osteoarthritis to study the effects of urolithins on inflammatory pain. The meniscotibial and medial collateral ligaments were transected in the knees of mice to induce osteoarthritis as inflammatory pain model. Mice were given a diet containing urolithins to identify the effects on pain. Results showed urolithins reduced pain hypersensitivity and slowed down disease progression in mice [155]. In clinical trials of older adults, supplementation with 1000 mg/day urolithin for 4 months showed no adverse events [156]. Additionally, plasma levels of inflammatory biomarkers were significantly reduced in a group that consumed urolithin. However, further experiments are needed to evaluate the effects of urolithins on pain relief. 

**Table 1:** Natural product-derived compounds attenuating microglial activation-mediated inflammatory pain.

Class of Phytochemicals	Subclass	Major Compound	Source	Targeting Inflammatory Mediators	Targeting Intracellular Signaling	Inducer in Animal Model	Safety Dosage in Clinical Study	Effects in Clinical Study	Reference
Phenolics	Phenolic acid	3,5-Dicaffeoylquinic acid	*Arctium* *lappa, aster yomena*	TNF-α, IL-1β, IL-6, MCP1, MCP3, iNOS, COX2	JAK2/STAT3, Autophagy	CFA			[24]
Phenolics	Phenolic acid	Chlorogenic acid		NO, iNOS, TNF-α	NF-κB	Carrageenan, Formalin	480 mg/day for 8 weeks	Improvements in neuronal function	[102,103,104,105]
Phenolics	Phenolic acid	Ferulic acid	*ferula* *asafetida*	TNF-α, iNOS	NF-κB, JNK	Formalin	1000 mg/day for 6 weeks	Anti-oxidant, anti-inflammation	[106,107,108,109].
Phenolics	Phenolic acid	6-gingerol	*zingiber* *officinale*	NO, iNOS, IL-1β, IL-6	STAT3	Acetic acid, Formalin, Carrageenan	20 mg/day for 12 weeks		[110,111,113]
Phenolics	flavonoids	Curcumin	*Curcuma* *longa*	NO, PGE2, iNOS, COX2, TNF-α, IL-1β, IL-6	NF-κB, MAPK, Nrf2	CFA	1200 mg/day for 6 days	Analgesic effects, anti-inflammation	[114,115,116,117]
Phenolics	flavonoids	Kaempferol	Tea, broccoli	NO, PGE2, iNOS, COX2, MMP-9, TNF-α, IL-1β, IL-6	NF-κB, JNK, ERK, p38	Formalin	50 mg/day for 4 weeks	Anti-inflammation	[118,119,120,121,122]
Phenolics	flavonoids	Quercetin		NO, iNOS, TNF-α	NF-κB, Nrf2, ERK	CFA	500 mg/day for 8 weeks	Analgesic effects, anti-inflammation	[123,124,125]
Phenolics	flavonoids	Formononetin	*Trifolium* *pretense* L.	TNF-α, IL-1β, IL-6, iNOS, COX2	NF-κB	CFA			[126,127]
Phenolics	flavonoids	Naringenin		TNF-α, IL-1β, iNOS	MAPK	Carrageenan, Capsaicin, CFA, PGE2	900 mg for a day		[129,130,131]
Phenolics	stilbenes	Resveratrol	grape	TNF-α, IL-1β, iNOS	NF-κB, Autophagy	CFA	500 mg/day for 90 days	Analgesic effects, anti-inflammation	[133,134,135]
Phenolics	lignan	Honokiol	*Magnolia* *officinlis*	NO, iNOS, TNF-α, IL-1β, IL-6	Autophagy	Carrageenan, CFA	50 mg/kg for a week		[136,137,138,139]
Non-phenolics	phthalide	Ligustilide	the roof of *Angelica* *sinensis*	NO, iNOS, COX-2, TNF-α, IL-1β, IL-6, MCP1	NF-κB	CFA, Acetic acid, Formalin			[140,141,142]
Non-phenolics	saponin	Glycyrrhizin	*Glycyrrhiza* *glabra*	NO, TNF-α, IL-1β, IL-6	NF-κB	CFA	450 mg/day for 4 weeks	Anti-inflammation	[144,145]
Non-phenolics	omega-3 fatty acid	Docosahexaenoic acid	Omega-3 polyunsaturated fatty acid	TNF-α, IL-1β, IL-6, MCP1, CCL3, CXCL10	p38	Carrageenan			[146]
Non-phenolics	monoterpene	Paeoniflorin	*Paeonia* *lactiflora*	TNF-α, IL-1β, IL-6	NF-κB	CFA	35.8 mg/day for 7 days		[148,149]
Non-phenolics	alkaloid	Sinomenine	*Sinomenium* *acutum*	NO, TNF-α, IL-1β, IL-6, MCP1	NF-κB, p38	CFA	40 mg/day for 3 months	Anti-inflammation	[150,151,152]
		Muscone	Musk	NO, TNF-α, IL-1β, IL-6	JAK2/STAT3	CFA			[89]
		Urolithins	Secondary metabolite	TNF-α, IL-1β, IL-6, iNOS, and COX-2	ERK, p38, and NF-κB	Surgery	1000 mg/day for 4 months	Anti-inflammation	[154,155,156]
		Muscone	Musk	NO, TNF-α, IL-1β, IL-6	JAK2/STAT3	CFA			[89]
		Urolithins	Secondary metabolite	TNF-α, IL-1β, IL-6, iNOS, and COX-2	ERK, p38, and NF-κB	Surgery	1000 mg/day for 4 months	Anti-inflammation	[154,155,156]

## 6. Methods

Reference lists were searched for articles published until 19–20 June 2023, using the keywords “Microglial activation”, “Neuroinflammation”, “Inflammatory biomarkers”, “Inflammatory pain”, “Chronic pain”, “Intracellular signaling”, “Natural products”, “Pharmaceuticals”.

In our search for this review, we applied no limits for country of origin or study design. Articles published in a language other than English were excluded. 

## 7. Conclusions

Inflammation in peripheral tissues can lead to the activation of microglia in the dorsal horn of the spinal cord, which is a significant contributor to neuroinflammation and inflammatory pain. Several studies have shown that natural products and their compounds have the ability to regulate microglial activation. Additionally, many studies have proposed that microglial activation-mediated inflammatory pain can be modulated using natural product-derived compounds. However, it is important to consider the possibility of drug–drug interactions (DDIs) when multiple drugs are consumed in combination, as these interactions can potentially affect the pharmacological effects of each drug [157]. In the case of muscone, a previous study reported that it reduced the hypnotic and analgesic effects of ketamine, which is a widely used anesthetic [158]. Taken together, although toxicological and pharmacological studies are required to determine their safety in humans, natural product-derived compounds are potential therapeutic candidates for the treatment of inflammatory pain.

## Data Availability

Not applicable.
